# Prostate-specific extracellular vesicles as a novel biomarker in human prostate cancer

**DOI:** 10.1038/srep30386

**Published:** 2016-08-09

**Authors:** Yong Hyun Park, Hyun Woo Shin, Ae Ryang Jung, Oh Sung Kwon, Yeong-Jin Choi, Jaesung Park, Ji Youl Lee

**Affiliations:** 1 Department of Urology, Seoul St. Mary’s Hospital, College of Medicine, The Catholic University of Korea, Seoul, Republic of Korea; 2 Catholic Cancer Research Institute, College of Medicine, The Catholic University of Korea, Seoul, Republic of Korea.; 3 School of Interdisciplinary Bioscience and Bioengineering, Pohang University of Science and Technology, Pohang, Republic of Korea.; 4 Department of Hospital Pathology, Seoul St. Mary’s Hospital, College of Medicine, The Catholic University of Korea, Seoul, Republic of Korea.

## Abstract

Extracellular vesicles (EVs) may play an important role in cancer development and progression. We aimed to investigate the prognostic potential of prostate-specific EVs in prostate cancer (PCa) patients. Plasma and prostate tissue were collected from patients who underwent surgery for PCa (n = 82) or benign prostatic hyperplasia (BPH, n = 28). To analyze the quantity of EVs in prostate, we performed transmission electron microscopy (TEM), immuno-TEM with CD63 and prostate-specific membrane antigen (PSMA), and immunofluorescence staining. After EV isolation from plasma, CD63 and PSMA concentration was measured using ELISA kits. PSMA-positive areas in prostate differed in patients with BPH, and low-, intermediate-, and high-risk PCa (2.4, 8.2, 17.5, 26.5%, p < 0.001). Plasma PSMA-positive EV concentration differed in patients with BPH, and low-, intermediate-, and high-risk PCa (21.9, 43.4, 49.2, 59.9 ng/mL, p < 0.001), and ROC curve analysis indicated that plasma PSMA-positive EV concentration differentiated PCa from BPH (AUC 0.943). Patients with lower plasma PSMA-positive EV concentration had greater prostate volume (50.2 vs. 33.4 cc, p < 0.001) and lower pathologic Gleason score (p = 0.025). During the median follow-up of 18 months, patients with lower plasma PSMA-positive EV concentration tended to have a lower risk of biochemical failure than those with higher levels of prostate-specific EVs (p = 0.085).

Prostate cancer (PCa) is the most common solid cancer in men in the United States with 233,000 new diagnoses and 30,000 cancer-related deaths in 2014^1^, and the fifth most common cancer in Korean men^2^. The clinical features range from a minute low-grade cancer that may be clinically insignificant to an aggressive high-grade cancer that ultimately causes progression to metastasis, castration-resistance, and death. Prostate-specific antigen (PSA) has become the most frequently used biomarker for PCa; however, considerable controversy remains regarding the use of PSA because of its low specificity and unclear relationship with stage and grade^3^. Thus, the development of new biomarkers is crucially needed for early detection of PCa and for prediction of prognosis and treatment response, in order to determine which patients require radical treatment versus active surveillance, and to identify those who would be most likely to respond to specific drugs.

Extracellular vesicles (EVs) have recently come into the spotlight with the understanding that these are not merely cell fragments^4^, but rather maps of their cells of origin with both physiological and pathological relevance^5^. EVs are nano-sized (30–120 nm in diameter) membrane-bound vesicles that are categorized into exosomes, microvesicles or ectosomes, and apoptotic bodies^6^,^7^. Accumulating evidence indicates that EVs may play an important role in cell-to-cell communication^8^,^9^ and cancer development and progression^10^. However, despite the overall prevalence and clinical importance of PCa, only a limited number of studies have indicated that EVs have prognostic relevance in PCa. Therefore, we aimed to investigate the levels of prostate-specific EVs isolated from the plasma of patients with benign prostatic hyperplasia (BPH) and PCa of different stage and grade and to evaluate the prognostic potential of prostate-specific EVs in PCa patients.

## Results

### Baseline demographics of the patients

For our cohort of 110 patients, the mean age of PCa patients was 67.5 years and the mean age of BPH patients was 72.7 years (p = 0.002). In BPH and PCa patients, the mean PSA level was 5.5 ng/mL and 12.9 ng/mL (p = 0.001) and the mean prostate volume was 68.6 cc and 34.6 cc (p < 0.001), respectively.

### Extracellular vesicles in prostate tissue

**Figure 1:**
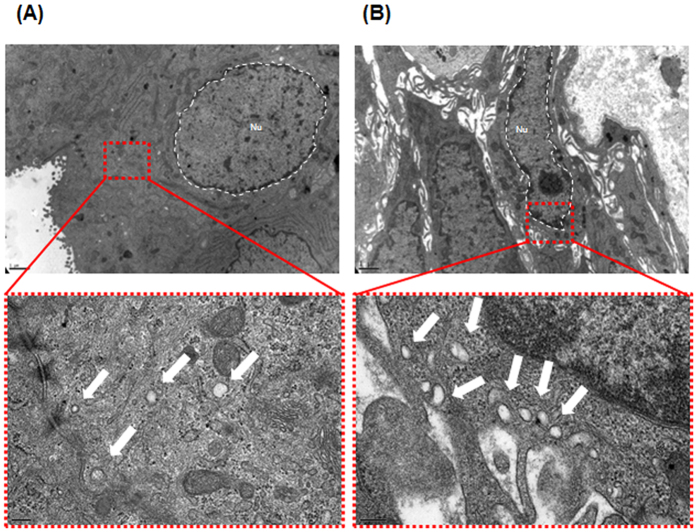
Representative transmission electron miscroscopy (TEM) images of extracellular vesicles (EVs) in prostate tissue. Vesicles 30–100 nm in diameter were observed by TEM. (**A**) Human benign prostatic hyperplasia (BPH) cells produce several microvesicles. The lower panel shows a magnified region of (A). The EVs appear as white dots (indicated by an arrow). (**B**) Human prostate cancer cells shed more microvesicles compared to BPH cells. The lower panel shows a magnified region of (B) Bars in low-magnification images, 1 μm. Bars in high-magnification images, 200 nm.

**Figure 2:**
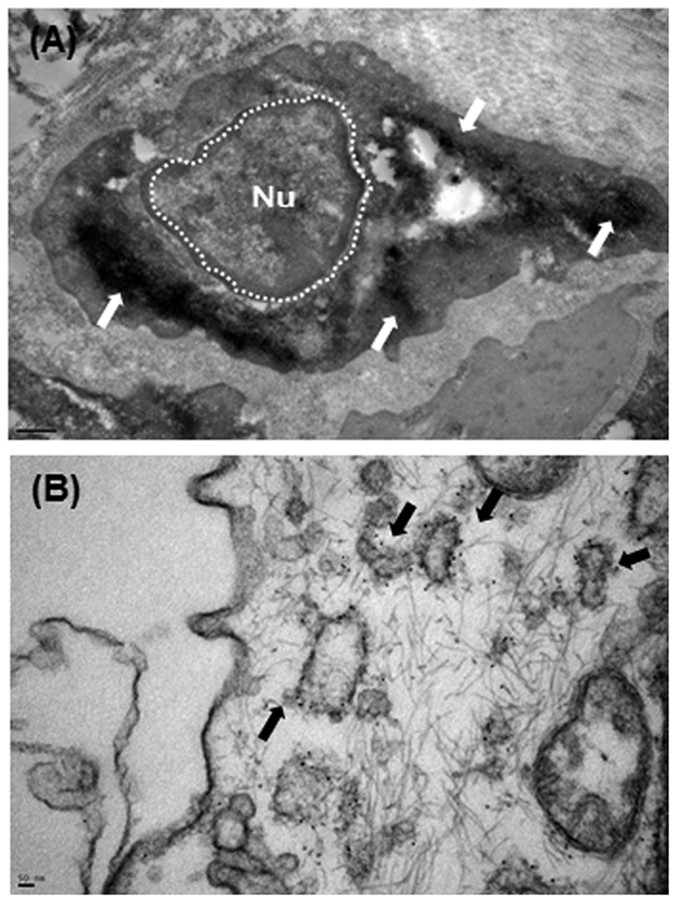
Representative TEM images of (**A**) immunoperoxidase/diaminobenzidine methods and (**B**) immunogold enhancement showing ultrastructural localization of PSMA. Bar in (A) 1 μm. Bar in (B) 10 nm.

As shown in Fig. 1, TEM revealed several vesicles that were mainly nanosized (30–100 nm in diameter) with the characteristic round shape of EVs in the cytoplasm of BPH (Fig. 1A) and prostate cancer tissues (Fig. 1B). The number of EVs observed by TEM was higher in prostate cancer cells than in BPH cells. In immuno-TEM with an anti-PSMA antibody, a recognized EVs marker^11^, the DAB deposits (Fig. 2A) and gold precipitations (Fig. 2B) were clearly recognized as diffuse dense profiles and fine dark particles, respectively, indicating the presence of PSMA within the vesicles.

**Figure 3:**
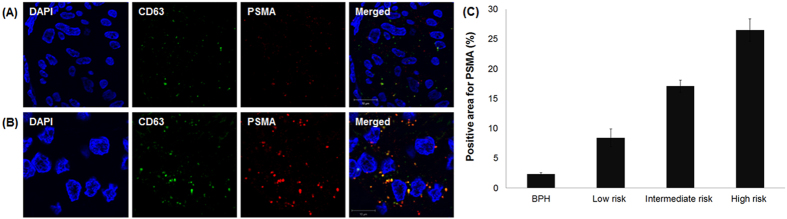
Representative images of immunofluorescence staining for CD63 and PSMA in patients with (**A**) benign prostatic hyperplasia and (**B**) prostate cancer. (**C**) Quantification of PSMA-positive areas in prostatic tissue (p < 0.001).

Analysis of prostatic tissue by confocal microscopy showed a punctate pattern of colocalized CD63 (green) and PSMA (red), confirming the TEM results (Fig. 3). The positive areas for PSMA were significantly different in patients with BPH, and low-risk, intermediate-risk, and high-risk PCa (2.4, 8.2, 17.5, 26.5%, respectively, p < 0.001).

### Extracellular vesicles in plasma

**Figure 4:**
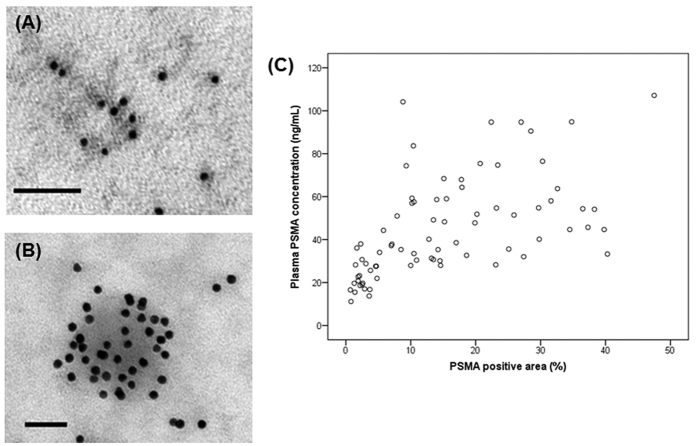
Representative images of TEM with immunogold enhancement with anti-CD63 (**A**) and PSMA (**B**) antibodies. (**C**) Correlation between the plasma PSMA-positive EV concentration and PSMA-positive areas in prostatic tissue (Spearman’s rho correlation coefficient = 0.672, p < 0.001).

**Figure 5:**
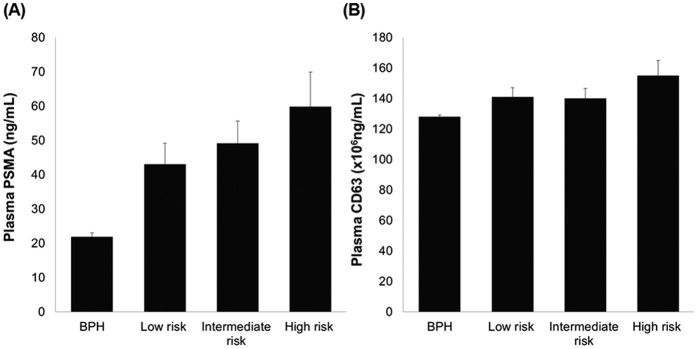
Quantification of the concentration of (**A**) plasma PSMA-positive EV (p < 0.001) and (**B**) plasma CD63-positive EV (p = 0.067).

**Figure 6:**
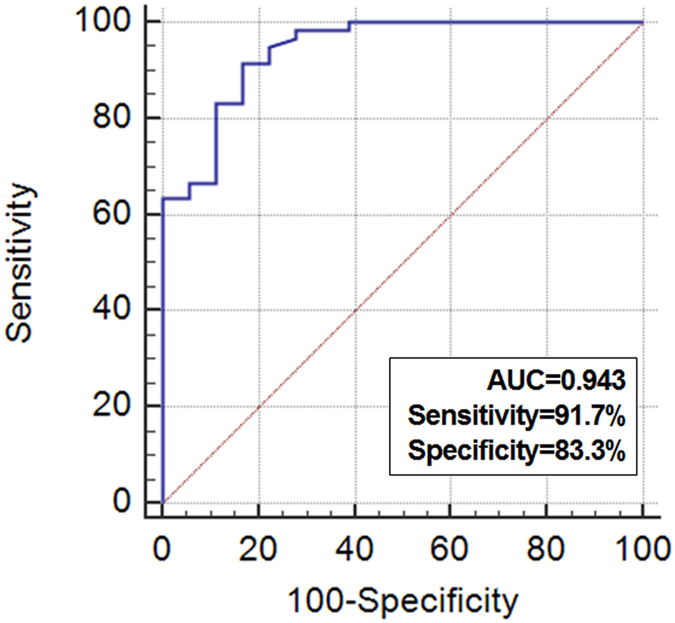
Receiver operating characteristic curve analysis using plasma PSMA-positive EV concentration for discrimination of prostate cancer from benign prostatic hyperplasia.

Because our main concern was to identify the usefulness of plasma EV concentration for liquid biopsy, we isolated EVs from the plasma of the patients. After the isolation process, the first step in our analysis was to establish whether we had successfully isolated EVs. TEM and immunogold-TEM analysis revealed many vesicles with a typical round shape and immunoreactivity of CD63 (Fig. 4A) and PSMA (Fig. 4B) in the plasma extracts. In BPH and PCa patients, the mean plasma PSMA-positive EV concentration was 21.9 ng/mL and 51.5 ng/mL (p < 0.001) and the mean plasma CD63-positive EV concentration was 128 × 10^6^ ng/mL and 145 × 10^6^ ng/mL (p = 0.067), respectively. Plasma PSMA-positive EV concentration showed good correlation with PSMA-positive areas in prostatic tissue (Spearman’s rho correlation coefficient = 0.672, p < 0.001; Fig. 4C). Plasma PSMA-positive EV concentration was statistically different among patients with BPH, and low-risk, intermediate-risk, and high-risk PCa (21.9, 43.4, 49.2, 59.9 ng/mL, respectively, p < 0.001), whereas plasma CD63-positive EV concentration was not significantly different among patients with different disease status (128, 141, 140, 155 × 10^6^ ng/mL, respectively, p = 0.114; Fig. 5). ROC curve analysis indicated that plasma PSMA-positive EV concentration was a valuable biomarker for differentiating PCa from BPH with excellent AUC (0.943, 95% CI 0.866–0.983; Fig. 6). At the cutoff value of 28.2 ng/mL for plasma PSMA-positive EV concentration, the optimal sensitivity and specificity were 91.7% and 83.3%, respectively.

### Clinicopathologic characteristics according to plasma prostate-specific EV concentration

**Figure 7:**
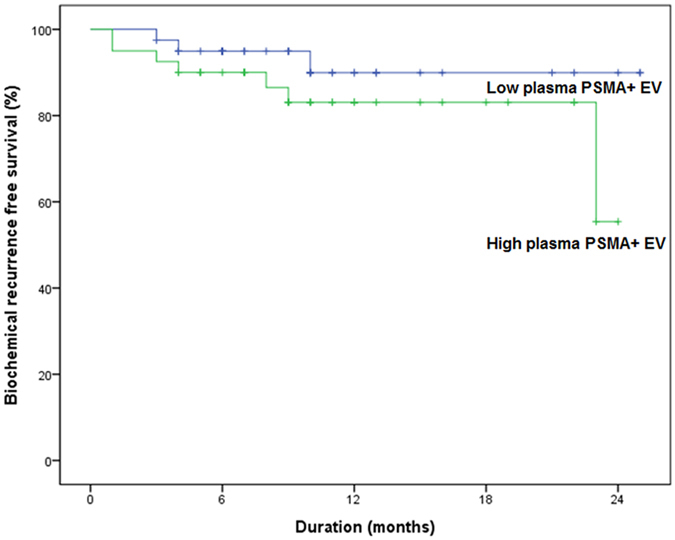
Biochemical recurrence free-survival according to plasma PSMA-positive EV concentration (p = 0.085).

Using the cutoff value of 28.2 ng/mL for plasma PSMA-positive EV concentration, patients were stratified into two groups: low EV and high EV group (Table 1). Patients with low EV level had lower preoperative PSA concentration (10.4 vs. 13.2 ng/mL, p = 0.095), and greater prostate volume (50.2 vs. 33.4cc, p < 0.001) than those with high EV levels. Also, patients with low EV had lower pathologic Gleason score (p = 0.025). However, there were no significant differences in pathologic T stage and tumor volume according to the plasma PSMA-positive EV concentration. During the median follow-up of 18 months, patients with lower prostate-specific EVs tended to have a lower risk of biochemical failure than those with higher prostate-specific EV (p = 0.085; Fig. 7).

## Discussion

Precision medicine relies on identifying which treatment options will be effective for individual patients based on their genetic, biologic, and lifestyle factors^12^. In the pursuit of this goal, tissue biopsy from primary or metastatic lesions is used to analyze molecular events, generally at a single time point. However, these biopsies have numerous challenges, including cost, potential morbidity of biopsies, and, most importantly, tumor heterogeneity. Given the complexities of tumor heterogeneity and molecular evolution during the duration of treatment, a tissue biopsy sample may not be a true representation of the molecular profile of the individual patient. Liquid biopsy may represent the final frontier of non-invasive methods to detect and monitor molecular characteristics of tumor and is currently used for circulating tumor cells and circulating tumor DNA^13^,^14^. However, this analysis is challenging because of the very low concentrations of analytes in the blood or urine and stringent technical quality control^13^. As EVs are present in increased number in malignant disease, and, moreover, can be easily recovered from biological fluids and resistant to metabolic processes, they might have potential as biomarkers for diagnosis, prognosis, and treatment response^15^.

In this study, we investigated plasma prostate-specific EVs in PCa patients. Unlike PSA screening or monitoring, which may not cancer-specific, we successfully demonstrated a difference in plasma prostate-specific EV concentration between BPH and PCa, together with differences in pathologic outcomes of PCa patients according to the plasma EV concentration. To date, only a few controversial studies have been conducted to examine the diagnostic or prognostic potential of EVs in PCa. In the early stages of EV research, Sahlén *et al.* reported that benign and malignant prostatic tissue show great similarities in the synthesis, storage, and release of EVs^16^. However, more recent research indicated great potential for EVs in the diagnosis and prognosis of PCa. Duijvesz *et al.* measured the urinary EV level after digital rectal examination using time-resolved fluorescence immunoassay and revealed that levels of EV markers, CD9 and CD63, were significantly higher in men with PCa^17^. Also, Huang *et al.* demonstrated that higher levels of exosomal miR-1290 and miR-375 were significantly associated with poor overall survival in patients with castration-resistant prostate cancer^18^. These recent results are consistent with our findings which demonstrated the potential clinical utility of EVs in identifying patients with high-risk of PCa.

So far, researchers have not taken advantage of EVs because of the lack of a standardized isolation method. Most of the isolation methods are labor-intensive and challenging due to co-isolation of contaminating non-EV materials, the failure to completely isolate EV fractions, or the loss of EVs due to damaged membrane integrity^19^. We successfully isolated EVs from plasma using an aqueous two-phase system with high recovery efficiency and in a short time (approximately 15 min)^20^. Aqueous two-phase systems have been used to separate particles that have different membrane surface properties with the advantages of scale-up potential, continuous operation, ease of process integration, low toxicity of phase forming chemicals, and biocompatibility^21^. In our previous study, we compared the EV recovery efficiencies of ultracentrifugation, ExoQuick^®^, and aqueous two-phase system and showed that the aqueous two-phase system recovered 68.3% of EVs from EV-protein mixture, whereas ultracentrifugation recovered only 15.2% and ExoQuick^®^ recovered only 38.8%. This method would allow easy and high-yield isolation of EVs in a short time without the need for specialized laboratory equipment.

Our study has several important strengths and weaknesses. We used the prospectively collected multicenter cohort sample of the Korea Prostate Bank, which is operated according to the best practices of the International Society for Biological and Environmental Repositories^22^. We evaluated the diagnostic and prognostic significance of prostate-specific EVs using these high-quality biological specimens under the evidence-based practices for collection, storage, retrieval, and distribution. Moreover, our study also confirmed the clinical usefulness of the aqueous two-phase system that could overcome the limitations of previous isolation methods, although this method requires standardization and external validation. However, we did not collect data reflecting long-term oncologic outcomes. Statistical insignificance of biochemical failure might result from the short follow-up, therefore, longer follow-up is required to confirm whether the trend for better biochemical recurrence is verified over the long term.

## Methods

### Patients

The study protocol was approved and carried out in accordance with the approved guidelines by the Institutional Review Board at the Catholic University of Korea, Seoul St. Mary’s Hospital (IRB approval No. KC14SISI0213). Plasma, fresh-frozen tissues, and paraffin-embedded tissues were supplied by the Korea Prostate Bank supported by the Korea Science and Engineering Foundation. To ensure a uniform cohort for evaluating plasma levels of EVs, patients were included only if they did not receive neoadjuvant androgen deprivation therapy, did not have a prior history of malignancy, and their plasma was obtained prior to surgery for PCa and BPH. Plasma, fresh-frozen tissues, and paraffin-embedded tissues from patients with low- (n = 17), intermediate- (n = 36), and high-risk (n = 29) PCa according to the National Comprehensive Cancer Network risk group^23^, and from patients with BPH (n = 28), were obtained from the Korea Prostate Bank with informed consent.

### Isolation of extracellular vesicles

EVs were isolated from plasma using a polyethylene glycol (PEG)/dextran (DEX) aqueous two-phase system^20^. Briefly, PEG (25–45 kDa, Sigma-Aldrich, St. Louis, MO, USA) and DEX (450–650 kDa, Sigma-Aldrich, St. Louis, MO, USA) were dissolved in PBS to give a PEG/DEX (21%/9% wt/wt) stock solution, and then 100 μl of the stock solution was added to 500 μl of plasma and the samples were vortexed. The samples were separated into two phases (DEX-rich and PEG-rich phase) by centrifugation at 1,000 × g for 10 min at 4 °C. During this process, EVs were effectively isolated into the DEX-rich phase because their surface interacted more strongly with DEX than with PEG. After phase separation, the DEX-rich phase was collected for further analysis by completely eliminating the PEG-rich phase.

### Morphology of EVs Using Transmission Electron Microscopy

The first step in our analysis was to determine whether EVs can be successfully observed in prostate tissue and plasma extract. Human prostate tissues and plasma extracts were fixed with 4% paraformaldehyde-2% glutaraldehyde in phosphate buffer (pH 7.4). The size and morphology of the particles were examined using transmission electron microscope (TEM), revealing vesicles with the typical size range (30~100 nm in diameter) and characteristic round shape of EVs.

To determine whether these vesicles were EVs, we performed TEM with immunoperoxidase/diaminobenzidine (DAB) methods and immunogold enhancement, which showed ultrastructural localization of CD63 and prostate-specific membrane antigen (PSMA). For immuno-DAB-TEM, samples were incubated with a blocking solution (1% bovine serum albumin in PBS) for 1 hour at room temperature and then with primary antibody against CD63 (diluted 1:100; Abcam, Cambridge, UK) or PSMA (diluted 1:100; Abcam, Cambridge, UK) overnight at 4 °C. After washing with PBS, the samples were incubated with secondary antibody for 1 hour at room temperature, rinsed briefly with PBS, and then visualized with a DAB kit (VECTOR, Burlingame, CA, USA). Cell nuclei were counterstained with hematoxylin and images were captured by TEM. For immunogold-TEM, EVs in prostate tissues and plasma extracts were fixed with 4% paraformaldehyde for 30 min at room temperature. The samples were washed three times with distilled water and then dropped onto formavarcarbon-coated grids and air dried for 10 min. The grids were blocked with 1% BSA for 20 min and incubated with primary antibody against PMSA or CD63 overnight at 4 °C (for the control the primary antibody was omitted). After washing, the grids were incubated with secondary antibody and images were captured by TEM.

### Immunofluorescence imaging of EVs in prostate tissue

Samples were incubated with a blocking solution (1% bovine serum albumin in PBS) for 1 hour at room temperature and then with primary antibody overnight at 4 °C. After washing three times with PBS, the samples were incubated with secondary antibody for 1 hour at room temperature and then mounted on slides. The slides were analyzed using a confocal microscope (LSM 510 Meta, Zeiss, Germany) equipped with ZEN 2009 Light edition software and an Olympus BX51 fluorescence microscope.

### Concentration of plasma EVs

EVs were isolated from human plasma and then suspended. The CD63 Exo ELISA Kit (EXOEL-CD63A-1, System Biosciences, Mountain View, CA, USA) was used for measurement of EV level according to the manufacturer’s instructions. PSMA level in EVs from human plasma was determined using the human glutamate carboxypeptidase 2 (FOLH1) ELISA kit (MBS901525, MY BioSource, Inc., San Diego, CA, USA) according to the manufacturer’s instructions.

### Outcome measurements and statistical analysis

Continuous variables are presented as mean (±standard deviation [SD]) and categorical variables are presented as proportions. Comparison of demographic, clinical and pathologic data was performed by the Student t-test or one-way analysis of variance (one-way ANOVA) test for continuous variables and the chi-square test for categorical variables. Correlations between PSMA-positive areas in the prostate tissue and the concentration of plasma PSMA-positive EVs were assessed using Spearman’s correlation coefficients (r). Receiver operator characteristic (ROC) curves and area under the ROC curve (AUC) were established to evaluate the diagnostic value of plasma PSMA-positive EV concentration for differentiating between BPH and PCa. Survival analysis for biochemical recurrence was performed using the Kaplan-Meier method, and differences between the groups were analyzed with the log-rank test. All p values were 2-sided, and data were considered statistically significant at p < 0.05.

## Conclusions

To the best of our knowledge, this is the largest report of the potential role of plasma prostate-specific EVs in differentiating PCa from BPH. Moreover, in patients with PCa, low concentration of plasma prostate-specific EVs was associated with favorable pathologic features and better biochemical recurrence-free survival.

## Additional Information

**How to cite this article**: Park, Y. H. *et al.* Prostate-specific extracellular vesicles as a novel biomarker in human prostate cancer. *Sci. Rep.* **6**, 30386; doi: 10.1038/srep30386 (2016).

## Figures and Tables

**Table 1:** Clinicopathologic characteristics according to plasma PSMA-positive EV concentration.

Variables	Overall	Plasma PSMA-positive EV concentration		
		Low	High	*p-value*
Diagnosis (%)				<0.001
BPH	28 (25.5)	28 (38.4)	0 (0)	
PCa	82 (74.5)	45 (61.6)	37 (100)	
Age (years)*	68.8 (±7.2)	69.7 (±7.2)	68.3 (±6.6)	0.406
BMI (kg/m^2^)*	23.3 (±2.6)	23.3 (±2.8)	23.3 (±2.7)	0.942
Preoperative PSA (ng/mL)*	11.0 (±16.7)	10.4 (±23.5)	13.2 (±13.9)	0.095
Prostate volume (mL)*,†	43.7 (±24.1)	50.2 (±23.1)	33.4 (±12.4)	<0.001
Pathologic T stage (%)				0.625
2	59 (71.7)	23 (76.7)	36 (69.2)	
3	22 (26.7)	7 (23.3)	15 (28.8)	
4	1 (1.7)	0 (0)	1 (1.9)	
Pathologic Gleason score (%)				0.025
≤6	19 (23.2)	12 (40.0)	7 (13.5)	
7 (3 + 4)	25 (30.5)	6 (20.0)	19 (36.5)	
7 (4 + 3)	23 (28.0)	9 (30.0)	14 (26.9)	
≥8	15 (18.3)	3 (10.0)	12 (23.1)	
PSMA-positive areas (%)	14.7 (±9.4)	8.1 (±8.7)	22.3 (±10.7)	<0.001

^*^Values are expressed as mean (±SD).

^†^Prostate volume was measured by transrectal ultrasonography.

BPH, benign prostatic hyperplasia; PCa, prostate cancer; BMI, body mass index; PSA, prostate-specific antigen; PSMA, prostate-specific membrane antigen.
