# Author Correction: Prostate-specific extracellular vesicles as a novel biomarker in human prostate cancer

**DOI:** 10.1038/s41598-019-41385-w

**Published:** 2019-04-15

**Authors:** Yong Hyun Park, Hyun Woo Shin, Ae Ryang Jung, Oh Sung Kwon, Yeong-Jin Choi, Jaesung Park, Ji Youl Lee

**Affiliations:** 1 Department of Urology, Seoul St. Mary’s Hospital, College of Medicine, The Catholic University of Korea, Seoul, Republic of Korea; 2 Catholic Cancer Research Institute, College of Medicine, The Catholic University of Korea, Seoul, Republic of Korea; 3 Department of Mechanical Engineering, Pohang University of Science and Technology, Pohang, Republic of Korea; 4 Department of Hospital Pathology, Seoul St. Mary’s Hospital, College of Medicine, The Catholic University of Korea, Seoul, Republic of Korea; 5 School of Interdisciplinary Bioscience and Bioengineering, Pohang University of Science and Technology, Pohang, Republic of Korea

Correction to: *Scientific Reports* 10.1038/srep30386, published online 09 August 2016

This Article contains errors in the Affiliations.

Hyun Woo Shin is incorrectly affiliated with ‘School of Interdisciplinary Bioscience and Bioengineering, Pohang University of Science and Technology, Pohang, Republic of Korea’. The correct affiliation is listed below:

Department of Mechanical Engineering, Pohang University of Science and Technology, Pohang, Republic of Korea

Additionally, this Article omits an affiliation for Jaesung Park. The correct affiliations for Jaesung Park are listed below:

Department of Mechanical Engineering, Pohang University of Science and Technology, Pohang, Republic of Korea

School of Interdisciplinary Bioscience and Bioengineering, Pohang University of Science and Technology, Pohang, Republic of Korea

